# The FeverApp registry – ecological momentary assessment (EMA) of fever management in families regarding conformity to up-to-date recommendations

**DOI:** 10.1186/s12911-020-01269-w

**Published:** 2020-10-01

**Authors:** David Martin, Jana Wachtmeister, Kai Ludwigs, Ekkehart Jenetzky

**Affiliations:** 1 School of Medicine, Faculty of Health, University of Witten/Herdecke, Alfred-Herrhausen-Straße 50, D 58448 Witten, Germany; 2 University Children’s Hospital, University of Tübingen, Tübingen, Germany; 3 Happiness Research Organisation, Düsseldorf, Germany; 4 Department of Child and Adolescent Psychiatry and Psychotherapy, University Medical Center Mainz, Mainz, Germany

**Keywords:** Fever, Children, Antipyretics, Registry, Ecological momentary assessment

## Abstract

**Background:**

Fever is one of the most common symptoms of pediatric consultations and its mismanagement is a health care burden. Guidelines on fever management are incoherent and data on fever management are still missing. This study protocol describes an app-based registry to evaluate the fever management of parents.

**Objective:**

The primary objectives are to assess guideline adherence (primary outcome) and parental confidence in managing fever, and thus to reduce overuse of antipyretics, antibiotics and healthcare providers.

Secondary objectives include creating a “FeverApp” that will enable parents to handle fever safely and to use the FeverApp registry as symptom and fever management diary. Further objectives include developing and testing a symptom-led registry model by app-based acquisition of parental entries of febrile illness cycle data and developing and testing models of how an interactive app-based registry can enable nationwide EMA information to inform science, guideline and policy makers, and the public.

**Methods:**

A FeverApp, guiding parents and carers in handling and documenting fever, will be developed with family pediatricians according to current guidelines and recommended for all parents in Germany. A registry will anonymously document features, management and outcomes of febrile episodes: basic sociodemographic and medical information, initial symptoms, course of fever, pharmacological and non-pharmacological interventions, consultations with doctors, outcomes, fever-associated fears, and app satisfaction.

**Results:**

This app may improve communication quality and health, e.g. asthma and antimicrobial resistance. Results will be published via website www.feverapp.de.

**Trial registration:**

This app-based registry protocol is registered in the German Clinical Trials Register (DRKS) with registration number: DRKS00016591.

## Introduction

### Relevance

We focus on the most common reason for pediatric consultation, the acute (viral or bacterial) febrile infection [1]. Across all levels of society and health care professionals there is a striking spectrum of conflicting views and approaches regarding fever management, leading to confusion, irrational fears and overuse of antibiotics and antipyretics [2, 3]. This is reflected by the plethora of opinions as found by a systematic review of all available official guidelines on how to handle fever from 195 countries that we performed during the conception phase. Recent recommendations based on systematic reviews no longer specify a temperature limit at which antipyretics should be applied [1, 4], while several older guidelines still do. The vast majority of recommendations are based on relatively weak evidence levels according to GRADE criteria (www.gradeworkinggroup.org). Even recommendations that have been made on the basis of a fairly high level of evidence have been slow to translate into clinical practice. As an example a Cochrane review including three randomized trials found no convincing evidence that antipyretic drugs prevent febrile seizures [5]. Nevertheless, antipyretics are still recommended by many doctors specifically for the prevention of febrile seizures.

Antipyretics and antibiotics have been used for decades, yet it seems that we as a society are still learning about when and how they should be used and are still uncovering new problems related to their uncritical use. The most prominent problems, antimicrobial resistance (AMR) and microbiome imbalance, are related to antibiotics, but there are also problems related to antipyretic use that have yet to be systematically investigated on a larger scale including their possible side effects on a short- term (e.g. hepatic injury [6], empyema [7–9], overdose [6], anaphylaxis [10, 11], midterm (e.g. anti- microbial resistance because antibiotics may be less effective when combined with antipyretics [12], asthma [13, 14]) and long-term (e.g. cancer [15, 16], ADHD when used in pregnancy [17]).

### Motivation

There is very little data on what exactly parents do when a child has a febrile episode and what the motivations behind these actions are. Interviews are hardly feasible on a large scale and do not necessarily reflect real-time actions and feelings. Although health insurance and medical records disclose prescriptions for specific diagnoses, they do not indicate:

1. the course of the febrile temperature and symptoms
2. which medications (over the counter and prescription) have actually been used and why
3. which (if any) alternative antipyretic methods (e.g., calf wraps/compresses) have been used and why, and
4. how the parents and child were feeling at the time.

Real-time data on home-based fever management would be a great asset in determining the workability of the current fever guidelines as well as guiding their further optimization. However, collection of real-time data via traditional investigatory methods would not only be highly uneconomical; it would undoubtedly influence parental behavior and autonomy. Information on the handling of fever should be captured promptly (since people rarely remember their own actions, motivations and feelings in detail) by the children’s caregivers themselves, as subjective attitudes, beliefs, and emotions influence adherence to guidelines.

Smartphones (used by ≈90% of parents under the age of 39 in Germany [18]) enable timely, direct and economic collection of data in the context of so-called Ecological Momentary Assessment (EMA) studies [19], allowing repeated sampling of subjects’ behavior in real-world, real-time settings, thus minimizing recall bias. In the German Google Play Store, eight “fever” apps can currently be found (each downloaded between 1000 and 100,000 times), which bears testimony to the high level of interest on the part of the parents in recording fever data by means of an app. To the best of our knowledge, a free app produced by doctors and scientists that: a) allows entry of data beyond temperature and type of medication, b) provides information on dealing with fever based on current scientific data and c) makes app data available to the scientific and medical community, does not exist. This is what we are designing.

The goal of the FeverApp Registry Study is to align scientific research with home-based fever treatment practices through the use of a two-way stream of information. A direct path of communication between medical research and parents will be created, whereby data regarding adherence to guidelines can be recorded and optimization of the guidelines based on this data can be directly reprogrammed into the app, increasing the speed at which research impacts clinical practice, and increasing the quality of parent-child-doctor-nurse relationships.

## Methods

### Research question and hypothesis

The primary objectives are to assess guideline adherence (primary outcome) and parental confidence in managing fever, and reduce overuse of antipyretics, antibiotics and healthcare providers. Hence to increase guideline adherence in the long term. Adherent behavior thereby refers to common febrile infection of an otherwise healthy child; other, much rarer, forms will be filtered out based on course and entered diagnoses, or statistically absorbed.

- Adherence behavior 1: “Antipyretics should, if at all, be used to reduce current – as opposed to anticipated – suffering and not with the sole aim of lowering the temperature or preventing simple febrile seizures. In a normal, otherwise healthy child with a febrile infection, there is no temperature above which fever must be suppressed” [1, 4]. Note that this may not apply to all children since up to now no study has tested this question at the scale we are expecting the FeverApp registry to reach – which is one of the reasons for the registry.

Non-adherence behavior 1 is therefore defined as: giving an antipyretic to a child with a good or neutral well-being rating.

- Adherence behavior 2: “Consultation with a practitioner should, after the age of three months, be prompted by the “red flag” symptoms” (warning signs as identified in the guidelines of the Association of Child and Adolescent Physicians Association in Germany (BVKJ) together with board members of the German Society of Pediatrics and Adolescent Medicine (DGKJ) and included in the app) and not merely by an increased temperature. We, therefore, define non-adherence behavior 2 as “consulting a doctor without having indicated red flag symptoms in the app” and “not seeking a doctors’ advice, although red flag symptoms had been entered”. This may, of course, happen for a number of very justified reasons, which can be selected or entered in free text, which will improve the next guideline production.

#### Secondary objectives

To create as ecological momentary assessment a FeverApp which enable parents to handle fever safely, and use it as a symptom and fever management diary, resulting in an anonymous FeverApp registry. Further, to develop and test a symptom-led registry model by app-based acquisition of parental entries of febrile illness cycle data and to develop and test models of how an interactive app-based registry can enable nationwide EMA information to inform science, guideline and policy makers, and the public (via a website showing descriptive statistics and a map).

In concrete terms the following information will be anonymously acquired and analyzed (subject to change as the registry model matures):

Amount and sequence/timing of

- temperature,
- other symptoms including febrile seizures,
- interventions (e.g. medication and physical interventions),
- physician/hospital contact
- parental perception of child wellbeing,
- parental confidence.

Interactions between these parameters and stratified analyses according to 1) gender, 2) age, 3) baseline existence of fever phobia, 4) region (postal code), 5) culture (language), 6) number of children. Nationwide longitudinal changes of health insurance data in the use of antipyretics and antibiotics during observation period.

Due to the size of the subgroups some stratified subgroup analyses may only be possible at later stages. In particular, what influence do age, gender, family size, mobile device model and socioeconomic, educational and cultural background have on guideline adherent behavior?

As well in the later part register-based RCTs (RRCTs) with the help of A/B testing. For instance, we hypothesize that an analysis of the data acquired on behavior in dealing with febrile events during the first registry part will lead to the uncovering of optimization potentials in the current guidelines. These can then be implemented in the later part of the registry. Introducing randomized A/B version testing of various materials in RRCTs [20] can lead to step-by- step evidence-based improvement of the information material and guidelines.

Febrile seizures: The current hypothesis is that febrile seizures are not preventable by any sensible means [5]. Yet there are indications that febrile seizures may in some cases be prevented by rapid warming of the child [21] (explained by the fact that some pyrogenic mediators like IL-6. IL-1 beta and TNF-alpha, which lower the seizure threshold [22], are suppressed when a person is warmed [23]. A RRCT giving special indications to a randomized subset of parents could provide clues as to whether this hypothesis has any validity.

### Recruitment and participants

The FeverApp can be downloaded in the App Store and Google Play. The FeverApp is only be available via individual access codes distributed by the pediatric practices in Germany during the routine check-up and vaccination visits. In total, 6300 pediatricians are invited in 2020 for this registry through the BVKJ for at least the next 5 years. Up to twelve reference practices will provide us with a validation sample in the substudy “Students in Reference Practices” (StuRP).

Eligible participants are all care givers of children (aged between 0 and 17 years) in Germany with knowledge of German, English, Russian and potentially other languages, who own a smartphone and are willing to document at least one febrile episode of their child with the FeverApp.

### Sample size

To analyze guideline-adherent and non-adherent behavior at a 95% binomial confidence interval with an accuracy of ±2%, at least 455 fever phases are required if the smaller group comprises 5% (95%-KI: 0.03–0.07). Our a-priory estimation based on surveys is that only about 15% [24] of caretakers have a guideline-conforming approach to the management of fever, hence 1224 fever events are needed for the planned accuracy. If adherent and non-adherent groups are approximately the same size (95%-KI: 0.48–0.52), a maximum of 2400 fever phases are required [25].

The exact incidence of fever per age group is not yet known. Larger samples are required – and expected – for subgroup analyses (e.g. between age groups). Recruitment will therefore continue throughout year 5 and beyond. The Chi^2^ test will be used to compare two subgroups. Sample size planning cannot take unplanned multiple testing into account; therefore the results can only be interpreted exploratively.

Subsample calculations: If a difference of 5% between two frequencies is interpreted as clinically significant, at a power of 80% and an alpha error probability of 5% assuming normal distribution in both subsamples, the following sample sizes are required: For a very rare sample, 190 cases per subsample are sufficient. If the rate is around 50%, 1605 cases per subsample are required.

A plausible and representative sample of the gender of the child and the region is a prerequisite for all analyses. All analyses will be regularly discussed with our clinical and scientific advisory boards (members of the Boards of the BVKJ, DGKJ and of the German Working Group of Pediatric Epidemiology, as well as patient representatives) to avoid underpowered and invalid analyses. Recommendations (e.g. STROBE Statement [26]) are applied.

### Research procedure

To ensure usability and required recruitment rate for the main outcome on the one hand and to validate patient entries against clinical records and evaluate participation representativity on the other hand, we will implement a soft launch of the FeverApp in the StuRP substudy: 12 medical students will be assigned to attend a Reference Practice for 4 weeks. After 3 months, 6 months, and then yearly, the students will visit their respective reference practice and speak with physicians, assistants and parents to assess these health professionals’ and parents’ level of satisfaction with the FeverApp and report their results to the project leader.

Validation and representativity through StuRP: All parents will be given an information sheet about the FeverApp Registry and will be asked whether they are willing to sign an informed consent allowing registry staff to collect additional data from their medical records. The records will be tagged to remind the doctors to document: diagnoses including febrile seizures, febrile illnesses and symptoms, prescriptions and taken medication, emergency or hospital presentations. It will be compared to the app data for completeness of recording febrile events via physicians and parents. Subject to consent by the parents, the registry staff will have the option to contact the parents via mail, post, and phone to enquire about further details. We estimate the enrolment of approximately 20 parents per day per practice leading to a total of 4800 (20 parents × 20 days × 12 practices) parents. Depending on the use of the app we expect documentation of at least 2400 febrile illnesses (assuming 50% of parents who download the app and sign informed consent report a febrile episode in the app within the next 2 years) – which is the number needed to estimate the guideline adherence.

After testing in our reference practices via the StuRP Project, a hard launch of the FeverApp will ensue. After downloading the app, a short introductory video about the FeverApp Registry Study will be shown, followed by a brief explanation of the most important functions of the app. Parents can then create their children’s profiles. This provides baseline data. Finally, parents are guided through the fever check- list, where they get to know the most important symptoms of fever.

When a child becomes ill, the parents open the app and enter data, guided by the checklist. The app registers a new febrile episode and gently reminds parents three times per day to enter data, as the BVKJ currently advise during febrile illness. It also allows parents to make entries or notes at will. The Fever Info library and the website will provide constantly updated information on fever management.

Every 3 months, a brief push message is sent to the parents, asking them about the child’s current state of health, any febrile episodes that have not yet been entered into the app, as well as medical and hospital presentations since the last use of the app.

### Data set

The app can be used by multiple family members with the same access code (n:1). Each app user can create different profiles for each child (1:n). A profile of a child has some basic data and receives regular reminders each 3 months, to prevent loss of follow-up documentation of events (fever events, symptoms and treatment), which can be entered at any time. A child profile may have multiple febrile events (1:n) during observation time. A fever event may have two to multiple symptom measurements according recommendations. These fever event data elements are based on current guidelines and research, reviewed together with our clinical and scientific advisory boards.

Fever events have at least two symptom and measurement points (start and end), but can also have multiple measurements, due to the three reminders per day in case of fever. These consist of several characteristics: such as temperature and respiration rate measurements, symptoms, action and drugs taken.

Six levels of data elements may be considered with 1:n relation: A) pediatric practices give access to app usage in B) families with one access code but C) multiple app installations (roles). Hence in each family (B) several roles (C) can observe D) multiple children (profiles) with ongoing documentation of E) fever events, each with multiple F) temperature measurements, symptoms, measures and drugs taken.

There are following lists, self-extended via the text entries of the user: G) symptoms, H) drugs, I) action taken. By default, the symptom list/grid has the relevant symptoms according to the guidelines – this can also be extended via the respective text entries of the user. The list of drugs consists of the complete catalogue for drugs available in Germany with central pharmaceutical number. The app reminds the user every 3 months to check forgotten entries and may announce further studies, such as registry based randomized controlled trials (RRCTs).

C) App user data: Installation, smartphone model, iOS/Android version, app version and the chosen language are automatically recorded when the app is started. Every user event or action is recorded. These items are required for quality assurance and user research. The time stamp can be used to investigate possible triggers for the download, e.g. were there more downloads in winter than in summer or was there a flu wave? The smartphone model can be used to make assumptions about social status and functionality on different operating systems. The number of recorded children profiles is as well a characteristic of the app user. To maintain high parental trust and privacy, only the postal code is requested as demographic family information.

Generic information: an audit trail of all manual app entries, including their exact date and timestamp will be stored.

D - Child profiles: When parents create a child profile they can choose an arbitrary name, internally we will merely apply a number. For each child only month and year of birth is recorded to calculate current age. The gender of the child is recorded in order to investigate possible gender differences; the history of fever has an influence on how fever is treated and how confident parents feel in dealing with it; the history of antipyretic and antibiotic use is recorded because it is relevant to the question of long-term complications and to the attitude of parents towards drugs; chronic diseases, such as asthma or allergies, are assessed because they may be possible consequences of antipyretic or antibiotic use. Finally, we ask about parental confidence regarding fever in this child, the current weight and height.

E – Fever events: Entries of measurement, symptoms, pharmaceutical and non-pharmaceutical measures and free notes are possible at any time. They can be supplemented by manual entry of date and time of occurrences, in case they were in the past. In case of an acute febrile infection, parents will be encouraged to use the fever check-list and thereby enter the requested data three times a day. A fever event stops when parents press the “child is healthy” button or after 48 h without any entries of symptoms, fever or medication.

F – Measurements (check list): All items on the fever checklist are based on the BVKJ guideline and can be used to determine when a medical consultation is necessary: general condition of the child, momentary subjective feeling of competence of the parents, drinking and eating behavior, diarrhea, skin rash, pain, body temperature, (G) measurement method, respiratory rate and medical consultations.

Time, date, quantity and reason for medications (H) administered since the start of the current febrile illness, other previous and present measures (e.g. external applications) can be recorded. The FeverApp can scan the barcode of all medicines available in Germany.

Follow-up data: Every 3 months the parents are asked by push message whether any of the following have occurred since the last entry in the app: febrile episodes and related antipyretic (or antibiotic) use, interim symptoms, consultations with doctors or emergency services, the general fever confidence.

As mentioned in E (febrile events), parents will be encouraged to use the FeverApp as a medical diary for the recording of all symptoms (G) including febrile seizures and treatment (I), and especially all medicines (H) which the child receives with the date. These catalogues (G,H,I) are based on recommended guidelines content, but can be individually supplemented.

### Statistical analysis

As primary analysis we calculate guideline adherence regarding use of antipyretic and regarding consultation of physician. This is a pure relative frequency with a binomial confidence interval, additionally stratified according to gender, age and region.

Secondary analyses: All results without sample size prespecification are considered as exploratory, but power calculations will be considered in the peer-review approved registry statistical analysis plan (SAP) a priori during the regular scientific advisory board meetings. This is especially important for subsample comparisons, which are performed mainly in a conservative manner with non-parametric tests. Only in case of clear normal distribution of depended variable, proved via Shapiro-Wilk test, may parametric procedures be applied. Further longitudinal change in time series analysis will be applied in the later existence of the registry.

For analyses of temperature development several values (duration, maximum temperature, gradient to maximum temperature, area under the curve; for total sample as well as stratified according to age, gender, postal code, symptom, and treatment modalities) will be calculated. Data validity in the StuRP will be a descriptive comparison of physician record documentation a posteriori and parental real-live documentation via FeverApp registry.

Descriptive data are reported as absolute and relative frequencies and 95% binomial confidence intervals. As a special feature, selected data are reported instantly on an internally accessible dashboard and, after verification, on a public website. This includes number of app users and numeric and graphic options flexibly stratified according to age, gender, crude location, distribution of temperature, heart rate and respiration rate and their quotients, maximum temperature in a child feeling well, relative frequency of parents feeling confident, proportion of antipyretic and antibiotic usage, doctor consultations per patient-day and relative frequency symptoms.

Qualitative analyses: Aspects of registry studies are hypothesis-generating, mainly descriptive and not confirmatory. However, they can have a high external validity and hence may supplement the knowledge of RCTs. They also provide the basis for future investigations. Therefore, the registry reports include qualitative analyses, which are not prespecified in the approved SAP.

## Results

During the conception phase, great importance was attached to the creation of a user-friendly presentation of an optimized current BVKJ and DGKJ fever guideline that can be included in the planned FeverApp. The BVKJ and DGKJ were, and will further be involved in both defining the specifications and information content of the app as well as in the production of informational material that will be presented in multiple forms (animated training video, info-texts and a checklist) via the app and have already been developed during the conception phase. Parents will be guided through the recommendations step-by-step and can simultaneously document the child’s condition in a structured manner. We have already improved the app in multiple user testings and made it available. We have the validation in a randomized manner in four StuRP practices. Now a larger dissemination in more practices is scheduled. Further, we established a website (www.feverapp.de) for registration of participating practices and information of parents.

## Discussion

To summarize, the goal of the FeverApp registry is to develop a tool from which parents and health care science may both benefit.

To date, there is no unitary fever management guideline as conflicting views predominate in this field – both scientifically and clinically. Real-time, home-based data including motivational reasons for actions of parents are still missing and would provide necessary insights for dissolving the controversies around fever management. An app-based approach is a promising way to assess such data while providing parents with the current state of recommendations – thus also implementing an action-based feedback loop to validate and optimize the recommendations.

Results of the registry are hence expected to further enhance scientific knowledge and guidelines. This can contribute to a safe, feasible guideline that reflects the current state of research and is adapted and personalized via an app to the respective cultural and educational context of the users, thereby giving parents the feeling of being able to deal with fever safely and competently. Increased confidence and decreased anxiety in dealing with fever should in turn lead to a reduction in the use of unnecessary antibiotics, antipyretics an emergency services, benefiting individual and population health.

The app-based data collection is transferable to other registries and offers advantages, such as a strong patient reference through direct survey, easy accessibility for large populations and low personnel costs, as one-on-one interviews are replaced or enhanced by the app. Depending on the question and test design, the inclusion of physicians by simple modifications of the app is conceivable. Communication with parents and patients can be one- or two-way. Data quality and consistency of the data can be checked directly by the app and, if necessary, requested again from the patient. Outliers or values that fall below or exceed the measurable limits can be identified directly by the app and parents immediately asked to recheck their entries. More detailed explanations can be easily inserted into the app (e.g. “Please do not round up the temperature value. A precise input of the temperature is very important.”) and, if necessary, hardware such as a Bluetooth thermometer can be coupled directly to the app to avoid input errors. Data collection via the app greatly reduces social desirability distortions compared to data gathering by a physician or study nurse. Parents and patients answer the questions in their own environment thus providing naturalistic, real-time results. The coherence and accuracy of the data are also increased, as the investigations are not suspended on weekends or public holidays. Finally, coding errors can be excluded as far as possible, as the coding is automated.

However, the usage of a health app comes with certain risks and challenges. According to Hussain [27], the most crucial challenges are those regarding quality. We therefore include scientific and clinical advisory boards in the process of content creation and regulation. In addition, the StuRP substudy is planned as an internal soft launch to evaluate outcomes and provide evidence of the effectiveness prior to public hard-launching of the FeverApp. Another challenge that has been highlighted by many researchers focuses on usability issues [27]. In addition to the professional advisory boards, we therefore also included patient representatives in the development process to make sure that the content is easy to understand for the target audience and that the overall navigation structure in the app reflects the user’s mental models. Although harm caused by health apps is rarely reported [28], the evaluation of other (health) apps showed possible negative psychological effects like depression triggered by self-monitoring [27] or decreased attention and productivity caused by the massive presentation of notifications [29]. We considered these risks in the development of the app by gathering as little data as necessary and using as little notifications as possible. In addition, we will evaluate those risks concerning fever management in the StuRP substudy.

A risk regarding the scientific feasibility is the regularity of app-usage. Incentives for a regular app usage are possible but, due to the very enthusiastic feedback from the doctors and parents surveyed so far and the high download numbers of similar apps, which offer significantly fewer functions, we do not assume that a monetary incentive is necessary. Monetary incentives lead to a crowding-out effect and a reduction in intrinsic motivation which results in test persons making less effort and evaluating an interaction more negatively [30]. A positive evaluation of the app is of particular importance to ensure its long-term use. If we do not achieve the planned sample size, we can intervene in StuRP-informed information campaigns via the network of our cooperating partner, the BVKJ.

## Data Availability

Data will be available through publication via website and papers and upon request with in scientific collaborations with the registry.

## Abbreviations

BMBF: Federal Ministry of Education and Research in Germany
BVKJ: Association of Child and Adolescent Physicians Association in Germany
DGKJ: German Scientific Society of Pediatrics and Adolescent Medicine
SAP: Statistical Analysis Plan
StuRP: ‘Student in Reference Pracitice’ a validation substudy
